# No advantage of laser-assisted over conventional intracytoplasmic sperm injection: a randomized controlled trial [NCT00114725]

**DOI:** 10.1186/1743-1050-3-5

**Published:** 2006-07-05

**Authors:** Kevin S Richter, Alana Davis, Jennifer Carter, Stephen J Greenhouse, Gilbert L Mottla, Michael J Tucker

**Affiliations:** 1 Shady Grove Fertility Reproductive Science Center, Rockville, MD, 20850, USA

## Abstract

**Background:**

Intracytoplasmic sperm injection (ICSI) is a component of infertility treatment often employed when conventional in vitro fertilization is unlikely to be successful. Despite good clinical results with ICSI, the procedure is typically associated with degeneration of a significant percentage (approximately 10%) of the treated oocytes. The cause of this degeneration remains unclear. Speculation that damage caused by oocyte compression during the injection procedure may be responsible has led to the development of a novel technique known as laser-assisted ICSI. This procedure involves drilling a small hole through the zona pellucida with a laser prior to sperm injection. Preliminary studies have suggested that laser-assisted ICSI may dramatically reduce oocyte degeneration rates. The objective of this study was to examine whether the reported benefits of laser-assisted ICSI could be verified on a larger, less-selected group of patients.

**Methods:**

Oocytes retrieved from 59 patients scheduled for ICSI were randomly divided into equal treatment and control groups. Oocytes in the treatment group were inseminated by laser-assisted ICSI, while oocytes in the control group were inseminated by conventional ICSI. Outcome variables (oocyte fertilization and degeneration, embryo cell numbers and fragmentation on days 2 and 3, and compaction and blastocyst formation rates) were compared between treatment and control groups by paired-sample *t*-test. Subgroup analysis was performed according to zona pellucida and oolemma breakage patterns.

**Results:**

No significant differences between treatment and control groups were observed for any of the measured outcome variables. However, fragile zonae pellucidae were associated with significantly poorer embryo quality, and fragile oolemmas that broke easily upon insertion of the injection needle were associated with a significantly higher oocyte degeneration rate. Nevertheless, there were also no between-treatment differences in clinical outcomes within these patient subpopulations.

**Conclusion:**

Contrary to previous reports based on smaller sample sizes, the results of this study suggest that there is no benefit of laser-assisted ICSI, either for the general population of ICSI patients, or for patients prone to zona pellucida or oolemma fragility.

## Background

Intracytoplasmic sperm injection (ICSI), a method of in vitro fertilization (IVF) in which a single sperm is introduced directly to the cytoplasm of a mature oocyte, has revolutionized the treatment of male factor infertility. Since the first reported pregnancies and births in 1992 [1], ICSI has become the treatment of choice for male factor infertility. In addition to this original indication, the use of ICSI has since expanded to a variety of other applications, such as when fertilization is reduced or absent following conventional in vitro insemination, when using cryopreserved sperm, and when preimplantation genetic diagnosis for monogenetic diseases is planned. Some authors have even advocated the generalized use of ICSI for all patients undergoing IVF [2]. Intracytoplasmic sperm injection was used in more than half of all fresh ART procedures using patients' own eggs performed in the United States during 2002 [3]. Given this widespread use of ICSI, any modifications that improve clinical outcomes of this procedure could benefit a large segment of the patient population.

Since its inception, ICSI has been performed by the mechanical penetration of the zona pellucida and the oolemma by a glass needle through which the sperm is injected into the cytoplasm. Although the effectiveness of this procedure has been clearly demonstrated [1,4,5], ICSI is typically associated with oocyte degeneration rates ranging from 5% to 19% [1,4,6-9]. The reasons for this oocyte degeneration are unclear. Observations of the zona pellucida of oocytes by scanning electron microscopy revealed no zona fragmentation during the procedure, and demonstrated that the injection hole closes immediately after the needle is withdrawn, with the injection site being nearly undetectable 15 minutes later [10]. Thus, zona damage does not appear to be the cause of ICSI induced oocyte degeneration. The mechanical compression and distortion of the oocyte that commonly occurs, to varying degrees, during injection is another potential source of damage leading to oocyte degeneration. Some oocytes may be more prone to damage from the ICSI procedure than others. For example, it has been reported that post-ICSI oocyte degeneration rates are significantly higher for oocytes with a fragile oolemma that breaks easily upon insertion of the injection needle than for other oocytes [6,7].

A novel approach of pre-drilling a hole through the zona pellucida with a laser prior to ICSI has recently been reported [11-14]. This alternative approach, called laser-assisted ICSI, allows the insertion of the sperm injection needle with less distortion of the oocyte and may therefore be less traumatic. Rienzi *et al*. [11] first reported a pregnancy using laser-assisted ICSI in a couple with four previous conventional ICSI failures and poor oocyte survival. They noted minimal oocyte deformation with the use of laser-assisted ICSI, and survival of 8 of the 13 metaphase II oocytes retrieved and injected. Two small randomized studies of patients with a previous history of high rates of oocyte degeneration (>20%) following ICSI, or who produced oocytes with fragile oolemmas, yielded dramatic statistically significant reductions in oocyte degeneration rates [12,15] (0.5% versus 16% and 2% versus 14%, respectively) and improved embryo quality [12] with the use of laser-assisted ICSI compared to conventional ICSI. Similar benefits have been claimed for piezoelectric ICSI [16].

The objective of this study was to evaluate the benefits of laser-assisted ICSI among unselected patients undergoing in vitro fertilization with ICSI. We describe results of the largest randomized trial examining laser-assisted ICSI yet reported, including comparisons of oocyte fertilization and degeneration rates, cell number and fragmentation on culture days 2 and 3, and compaction and blastocyst formation rates.

## Methods

Patients were enrolled and treated following discussion of the study and informed written consent between March and September of 2004. The Western Institutional Review Board (WIRB) approved the study protocol (WIRB Pro. Nr. 20040267).

Indications for ICSI were diagnosis of moderate to severe male factor infertility or a history of poor conventional insemination. All patients undergoing IVF with ICSI at Shady Grove Fertility Reproductive Science Center during the study period were eligible for participation in the study, limited to one cycle per patient. The only exclusion criterion was retrieval of fewer than six mature oocytes. Mature (Metaphase II, or MII) oocytes from each participating patient were divided into two equal groups. Half of each oocyte cohort was assigned to the treatment group while the other half was assigned to the control group. Group assignments were allocated according to a computer-generated randomized series, were kept in sealed envelopes and were unknown to embryologists until after division of the oocyte cohorts. Treatment assignments were coded on embryo culture dishes, but effective blinding of embryologists recording fertilization outcomes and embryo development was not possible, because the hole in the zona produced by the laser was sometimes visible.

Laser-assisted ICSI was performed on oocytes assigned to the treatment group using a 1.48 micrometer wavelength infrared laser (Zilos Laser System, Hamilton Thorne Research, Beverly, MA). We attempted to replicate the procedure described in previous reports [11,12] as closely as possible. A small diameter (5–6 micrometer) channel was drilled through most of the thickness of the zona pellucida using 3 to 5 low energy pulses at less than two microseconds pulse duration; the sperm injection needle was then passed through this channel. Conventional ICSI was performed according to standard protocols [17] without laser pre-drilling. Within individual patients, a single embryologist performed ICSI on all mature oocytes in both treatment groups. For oocytes inseminated by conventional ICSI, the zona pellucida was graded as normal, fragile, or difficult according to breakage characteristics when the injection needle was inserted. Oolemma breakage patterns (normal, fragile, or difficult) were recorded for oocytes in both treatment groups. Subgroup analyses were planned based on these zona and oolemma characteristics.

The primary outcome measure was the percentage of oocytes that degenerated following the ICSI procedure, as this was considered the most direct indicator of ICSI-induced damage. Degenerating oocytes were distinguished by dark necrotic cytoplasm and vacuolization at the time of examination for fertilization, 16–18 hours after ICSI, when the number of 2 pronucleus (2pn) oocytes was also recorded. Numbers of cells per embryo and degree of fragmentation (percentage of total embryo volume) were recorded on days 2 and 3 of in vitro culture. Embryos were transferred to patients on either day 3 or day 5 depending on the number and quality of embryos available. Non-transferred embryos were monitored through day 7 for compaction and blastocyst formation.

Total numbers of oocytes and embryos are reported in the tables along with the per-cycle means for clinical outcomes. However, it must be emphasized that valid analysis of treatment effects for this experimental design is based on the per-cycle means, not outcomes per oocyte or embryo. It is well-known that oocyte and embryo characteristics are much more variable among patients than within oocyte cohorts. Thus, comparison of outcomes per oocyte or per embryo violates the assumption of independent observations. In this respect, the conclusions of the first randomized study [12] may be questioned because the results were evaluated by X^2 ^analysis using individual oocytes, rather than patients, as the unit of analysis. The statistical test used in the other prior randomized study [15] was not reported, but p-values are also consistent with per-oocyte X^2 ^analysis of the data. Valid estimates of either the size of, or the p-value for, the treatment effects from these two previous studies are not available.

Paired-sample two-tailed *t*-tests were used to compare oocyte fertilization and degeneration rates, numbers of cells and degree of fragmentation on days 2 and 3, compaction and blastocyst formation rates, and numbers of embryos transferred between conventional and laser-assisted ICSI treatments. The enrollment goal was set at 60 patients, approximately twice the size of the only previous randomized study published at the time [12]. Power analysis was conducted according to Bausell and Li [18]. Previous examination of oocyte degeneration among patients undergoing ICSI at our center [19] provided estimates of the expected mean (10%) and standard deviation (14%) for oocyte degeneration rates. Based on these parameters and assuming no correlation between treatment and control outcomes within patients (i.e. *r *= 0), this sample size would provide a minimum of 80% power to distinguish a reduction in oocyte degeneration with laser-assisted ICSI of 7.3% (i.e. 2.7% versus 10%), less than half the difference reported by Abdelmassih *et al*. [12]. Any correlation between the treatment and control groups within patients would increase statistical power. For example, a moderate correlation of *r *= 0.4 would result in 95% power to distinguish the above difference, and 80% power to distinguish a reduction in oocyte degeneration of 5.6%.

## Results

A total of 80 candidates were enrolled following informed written consent. Twenty-one of these patients were subsequently excluded from the study because fewer than six mature oocytes were retrieved. From the remaining 59 patients, 775 mature oocytes were retrieved and inseminated in accordance with the experimental design.

Patient age ranged from 22 to 43 years (mean = 34.3 ± 4.0 SD). The majority of couples were diagnosed with male factor infertility alone (34/59, 58%). Other diagnoses included endometriosis (n = 4), tubal factor (n = 3), PCO (n = 3), diminished ovarian reserve (n = 2), ovulation disorder (n = 2), and unexplained infertility (n = 7). The remaining four couples were diagnosed with a combination of both male and female factor infertility. The overall mean fertilization rate was 71.1%, with a mean oocyte degeneration rate of 12.0%. Mean embryo cell number was 3.0 on day 2 and 5.6 on day 3. Mean degree of embryo fragmentation was 7.6% on day 2 and 9.8% on day 3. Embryos were transferred to 44 patients at the cleavage stage on day 3 (mean = 2.5 embryos per transfer), and to 14 patients at the blastocyst stage on day 5 or 6 (mean = 1.9 embryos per transfer). In only one cycle was no transfer performed due to poor embryo quality. There were 26 pregnancies (44.8% per transfer) with 34 implantations (24.5% per embryo), based on ultrasound detection of fetal cardiac activity five to six weeks after embryo transfer, resulting in 20 live births and 3 spontaneous abortions, with three pregnancies ongoing at last report.

Clinical outcomes are compared between laser-assisted and conventional ICSI in Table 1. No significant differences were observed for any of the measured outcome variables. The difference in blastocyst formation rates among the 14 cycles with blastocyst stage embryo transfer was nearly statistically significant at an alpha of 0.05. However, given that this probability estimate does not include an adjustment for multiple comparisons (thus increasing the potential for type I error), and none of the many other comparisons that were made approached statistical significance, we believe this difference was most likely due to random chance.

**Table 1:** Mean clinical outcomes per cycle compared between laser-assisted and conventional ICSI of mature (MII) oocytes, including within-patient correlation coefficients (*r*).

	Laser-assisted ICSI	Conventional ICSI	*r*	Treatment effect (95% CI)	P-value
Mature oocytes	6.6 (388)^a^	6.6 (387)^a^			
Percent 2pn	71.0 (276)^a^	71.3 (278)^a^	0.39	-0.3 (-7.2–6.6)	0.94
Percent oocyte degeneration	11.4 (43)^a^	12.6 (44)^a^	0.33	-1.2 (-6.2–3.8)	0.63
Cell number, day 2	3.0	2.9	0.46	0.1 (-0.1–0.3)	0.51
Percent fragmentation, day 2	7.0	8.4	0.57	-1.4 (-3.6–0.9)	0.23
Cell number, day 3	5.7	5.5	0.48	0.2 (-0.2–0.5)	0.38
Percent fragmentation, day 3	9.0	10.6	0.64	-1.6 (-4.0–0.7)	0.16
Embryos/ET, day 3, n = 43	1.4 (60)^a^	1.2 (52)^a^	-0.46	0.2 (-0.2–0.6)	0.32
Embryos/ET, blast, n = 14	1.2 (17)^a^	0.7 (10)^a^	-0.74	0.5 (-0.2–1.2)	0.15
Percent compaction^b^	46.5 (121/216)^a^	54.9 (137/226)^a^	0.49	-8.3 (-20.2–3.6)	0.17
Percent blastocyst^b^	28.7 (78/216)^a^	33.1 (83/226)^a^	0.28	-4.4 (-15.4–6.7)	0.43
Percent compaction, blast ET	90.3 (84/93)^a^	89.5 (76/85)^a^	0.32	0.7 (-7.2–8.7)	0.84
Percent blastocyst, blast ET	65.1 (61/93)^a^	51.7 (48/85)^a^	0.57	13.5 (-0.1–27.0)	0.051

^a ^Numbers in parentheses are total number of oocytes or embryos.

^b ^Compaction and blastocyst formation rates based on all embryos that were not transferred on day 3.

Zona pellucida and oolemma breakage characteristics were recorded for 50 of the treatment cycles. Normal zona breakage was observed in all oocytes inseminated by conventional ICSI in 19 of these cycles. The zona was difficult to puncture in some (11–80% per cycle, mean = 37%) of the inseminated oocytes in 23 treatment cycles. The zona was unusually fragile in some (14–50% per cycle, mean 24%) of the inseminated oocytes in 6 cycles. Clinical outcomes were compared between laser-assisted and conventional ICSI within each of these three subgroups of patients (Table 2). No differences between the two ICSI treatments approached statistical significance. In an additional two cycles (not included in the table) oocytes exhibiting difficult zona breakage and oocytes with fragile zonae were both observed.

**Table 2:** Comparisons of laser-assisted versus conventional ICSI outcomes according to zona breakage characteristics.

	Normal zona (n = 19)		Difficult zona (n = 23)		Fragile zona (n = 6)	
	
	Laser	Conv	Laser	Conv	Laser	Conv
Percent 2pn	69.5 (71/106)^a^	62.9 (68/109)^a^	73.9 (125/167)^a^	75.6 (120/161)^a^	80.2 (30/38)^a^	79.4 (32/39)^a^
Percent oocyte degeneration	11.6 (13/106)^a^	16.1 (16/109)^a^	10.7 (18/167)^a^	9.6 (16/161)^a^	9.4 (3/38)^a^	12.5 (4/39)^a^
Cell number, day 3	5.6	5.5	6.0	5.6	5.1	4.8
Percent fragmentation, day 3	10.7	11.0	7.3	7.1	15.7	21.1
Percent compaction	43.2 (19/46)^a^	59.2 (29/50)^a^	57.5 (74/109)^a^	64.2 (69/102)^a^	29.2 (4/20)^a^	28.8 (9/26)^a^
Percent blastocyst	19.3 (7/46)^a^	30.9 (12/50)^a^	39.6 (52/109)^a^	44.7 (47/102)^a^	20.8 (3/20)^a^	17.9 (6/26)^a^

^a ^Numbers in parentheses are total number of oocytes or embryos.

When the eight cycles with at least one oocyte with a fragile zona were grouped, the degree of embryo fragmentation on both days 2 and 3 was significantly greater than in cycles without any oocytes with a fragile zona (14.2% versus 6.8%, P = 0.018 and 17.0% versus 8.8%, P = 0.029 respectively, *t*-test). These cycles including oocytes with fragile zonae also exhibited significantly lower rates of compaction (23.8% versus 51.5%, P = 0.036), with a reduced blastocyst formation rate that also approached statistical significance (15.3% versus 31.8%, P = 0.084). No other outcome variables were significantly different between cycles with and without oocytes with fragile zonae.

Rates of oocyte fertilization and degeneration, and cell numbers and degree of fragmentation on days 2 and 3, did not differ significantly between cohorts including oocytes with difficult to penetrate zonae pellucidae and cohorts in which all oocytes had normal zonae.

Among all the oocytes for which oolemma breakage patterns were recorded, 25.9% had fragile oolemmas, while 22.7% had oolemmas that were difficult to penetrate. Both types of oolemma breakage patterns often occurred in the same oocyte cohort. The percentage of difficult oolemmas within a cohort was not significantly related to any observed clinical outcome. However, linear regression analysis indicated a significant positive relationship between the percentage of fragile oolemmas in a cohort and percent oocyte degeneration (P = 0.0026), and a significant negative relationship between the percentage of fragile oolemmas and percent fertilization (P = 0.0033). The oocyte degeneration rate among cycles (n = 24) with greater than 30% fragile oolemmas within the oocyte cohort was twice as high as that of cycles (n = 26) with less than 30% fragile oolemmas (17.0% versus 8.1%, P = 0.024, *t*-test). The percentage of fragile oolemmas was not significantly related to any other clinical outcome. Oocyte degeneration rates for laser-assisted relative to conventional ICSI were not related to the proportion of fragile oolemmas in an oocyte cohort (P = 0.14, linear regression).

Inseminations were performed by one of two embryologists (AD or JC) for the majority of treatment cycles (n = 29 and 23, respectively). Evidence of a learning curve was sought by linear regression analysis of the difference between laser-assisted and conventional ICSI outcomes according to the case number for each of these two embryologists. There was no evidence of significant change in relative outcomes over time for either embryologist (fertilization rate P = 0.74 and 0.30, degeneration rate P = 0.25 and 0.88, day 3 cell number P = 0.36 and 0.59, and day 3 fragmentation P = 0.97 and 0.68, respectively).

Transferred embryos were exclusively from the laser-assisted ICSI treatment group in 12 cycles, resulting in six pregnancies with four live births and two pregnancies ongoing at last report. Transferred embryos were exclusively from the conventional ICSI treatment group in eight cycles, resulting in six pregnancies with four live births and 2 spontaneous abortions. The remaining 38 transfers included embryos from both treatment groups, resulting in 14 pregnancies with 12 live births and one spontaneous abortion, with one pregnancy ongoing at last report. Among all 26 cycles resulting in pregnancy, 31 of the 60 embryos transferred (52%) were derived from laser-assisted ICSI. Among the 32 cycles without pregnancy, 46 of the 79 embryos transferred (58%) were derived from laser-assisted ICSI. A X^2 ^comparison indicated that embryos derived from laser-assisted ICSI were similarly represented in the pregnant and non-pregnant cycles (P = 0.44).

## Discussion

We found no evidence that pre-drilling through the zona pellucida prior to insertion of the ICSI needle improves oocyte survival or embryo quality, contrary to the conclusions of two smaller prior studies [12,15]. Rates of oocyte degeneration and fertilization, cell numbers and fragmentation rates on days 2 and 3, and rates of compaction and blastocyst formation were similar regardless of ICSI treatment type. The 95% confidence interval for a treatment effect on oocyte degeneration rates suggests that even if degeneration rates are lower with laser-assisted compared to conventional ICSI, the reduction is unlikely to be greater than 50%, much less than that suggested by the earlier studies.

However, despite being well designed, evaluation of the results of these two previous randomized studies is problematic because both used individual oocytes rather than patients as the unit of analysis. These 'unit of analysis' errors are common – a recent systematic review [20] identified 'unit of analysis' errors in 82% of clinical trials examined. The use of multiple observations per patient results in unpredictable bias in treatment effects estimates, and an exaggerated apparent sample size leading to spuriously low p-values and narrow confidence intervals [20].

Patient selection may have played a role in the difference between our results and those of the earlier reports. The earlier reports included patients selected based on a history of ICSI with high rates of oocyte degeneration [12] or fragile oolemmas [15], while ours was based on unselected patients undergoing ICSI. It is therefore possible that a subset of patients may benefit from laser micromanipulation, even if there are no significant benefits to the general patient population. However, the observed rate of oocyte degeneration in our control group was only slightly lower than reported rates of oocyte degeneration in the control groups of these earlier studies.

Characteristics of the zona pellucida, or possibly the oolemma, may indicate patients more likely to benefit from laser-assisted ICSI. It is well known that breakage patterns of the zona and oolemma can differ greatly between oocytes. Oocyte compression during the ICSI procedure is greatest in those eggs with zonae or oolemmas that are particularly difficult to penetrate. Laser drilling through a difficult zona pellucida eliminates the dramatic compression of the oocyte that would otherwise result from ICSI of such eggs. Fragile oolemmas have been reported to be associated with higher rates of oocyte degeneration [6,7]. However, it is more difficult to imagine a benefit of laser-assisted ICSI for a fragile oolemma because the oolemma is not contacted or manipulated by the laser during the procedure.

We attempted to evaluate the possibility of a benefit within such subgroups by subdividing our patient population according to the zona or oolemma breakage patterns of their oocytes. Although many patients who were included in this study exhibited zonae with difficult breakage, resulting in dramatic compression during injection without laser pre-drilling, we found no evidence of increased degeneration rates or poorer embryo quality among these oocytes, and no evidence that they benefited from the use of the laser. Embryos resulting from cycles with oocytes with fragile zonae exhibited a higher percentage of fragmentation and a reduced rate of compaction compared to cycles without fragile zonae, but laser-assisted ICSI did not improve these results.

Blastocyst formation rates appeared to be higher when oocytes with difficult to penetrate zonae pellucidae were present in the cohort compared to cohorts in which all zonae were normal. However, we believe this apparent relationship is a statistical artifact resulting from the fact that a much greater proportion of oocytes were transferred on day 3 in the normal zona group compared to the difficult zona group (31% versus 14%). Thus, calculations of compaction and blastocyst formation rates for embryos not transferred on day 3 (as reported in the tables) include proportionally fewer of the higher quality embryos in the normal zona group compared to the difficult zona group. In addition, it is biologically implausible that the presence of oocytes with abnormal zonae pellucidae would be associated with better embryonic development than when all oocytes within a cohort have normal zonae.

There were also many patients whose oocytes had fragile oolemmas, and many patients whose oocytes had difficult to penetrate oolemmas. Oocyte degeneration rates increased significantly with increasing proportions of fragile oolemmas, which is consistent with previous reports [6,7]. However, oocyte degeneration rates for laser-assisted relative to conventional ICSI did not decrease as the proportion of such oocytes within a cohort increased, indicating that laser-assisted ICSI did not reduce the high rates of degeneration associated with fragile oolemmas. Clinical outcomes were unrelated to the proportion of oocytes with difficult oolemma penetration within a cohort.

We do not believe that operator experience was a factor in our inability to distinguish a beneficial effect of laser micromanipulation. The lack of any detectable learning curve supports this contention. All embryologists performing ICSI in this study were highly skilled and had years of experience performing ICSI and working successfully with the laser for embryo manipulation procedures such as assisted hatching and embryo biopsy. Embryologists performing the procedure felt that it was relatively simple and could be performed quickly, although even with practice the entire process of insemination was still more time consuming with the addition of the laser micromanipulation. They also agreed that the compression of the oocytes during the ICSI procedure was dramatically reduced in the laser-assisted group, as previously reported [11].

We question the rationale for hypothesizing reduced oocyte degeneration with laser-assisted ICSI versus conventional ICSI. While laser-assisted ICSI does dramatically reduce oocyte compression, we are aware of no evidence suggesting increased oocyte degeneration in association with oocyte compression resulting from either zonae pellucidae or oolemmas that are difficult to penetrate, and our results do not suggest such a relationship. The one feature that has been clearly associated with oocyte degeneration rates, a fragile oolemma, seems unlikely to be responsive to laser pre-drilling of the zona pellucida because the oolemma is not manipulated by this procedure.

Although we found no clinical benefit to drilling through the zona with a laser before sperm injection, we also found no evidence of harm as a result of the laser micromanipulation of oocytes. Oocytes inseminated by laser-assisted ICSI were as likely to be fertilized successfully as, and had similar preimplantation developmental potential to, oocytes inseminated by conventional ICSI. This study therefore provides evidence for the safety of laser micromanipulation of human oocytes via the comparable preimplantation development of embryos derived from both the study and control groups.

## Conclusion

In conclusion, the results of this study suggest that there is no benefit of laser pre-drilling of the zona pellucida over conventional ICSI with regard to fertilization and embryo development. Moreover, examination of results according to the breakage patterns of the zona or the oolemma did not reveal any subgroups of patients that appeared to benefit from laser-assisted ICSI. Given the increased time required to add laser pre-drilling to the ICSI procedure, there appears to be no justification for its use. However, the potential for benefits within particular patient subgroups deserves further study. There was also no evidence that the laser caused damage that reduced preimplantation developmental potential.

## Competing interests

The author(s) declare that they have no competing interests.

## Authors' contributions

AD and MT conceived the study. AD, MT, KR, and SG designed the study protocol. AD and JC performed ICSI treatments and collected data. KR performed the statistical analysis. KR, SG and GM drafted the manuscript. All authors read and approved the final manuscript.
